# *Paraburkholderia* Mediates Salt Stress Alleviation in Cucumber Seedlings

**DOI:** 10.3390/plants15071104

**Published:** 2026-04-03

**Authors:** Xinyu Jia, Bin Tian, Jingwen Li, Shanyu Li, Mengxin Chen, Sai Wang, Yonghui Zhao, Lin Hao, Wei Fu

**Affiliations:** College of Life Science, Shenyang Normal University, Shenyang 110034, China; 13840290716@163.com (X.J.); tb15640464712@163.com (B.T.); 13898757428@163.com (J.L.); 15524253653@163.com (S.L.); mxchen0110@163.com (M.C.); wangsai0616@synu.edu.cn (S.W.); zhaoyonghui2020@163.com (Y.Z.)

**Keywords:** growth-defense tradeoffs, abiotic stress, ionic and osmotic adjustment, plant growth-promoting rhizobacteria, photosynthesis, phytohormone

## Abstract

To elucidate the cooperative regulatory mechanisms underlying *Paraburkholderia* sp. GD17-mediated salt tolerance in cucumber plants. Hydroponically grown cucumber plants were inoculated with GD17 and subsequently subjected to NaCl treatment. Physiological, biochemical parameters, as well as gene expression profiles, were comprehensively analyzed. GD17 inoculation significantly improved plant growth, developmental performance, and salinity tolerance. Under salt stress, GD17-inoculated plants exhibited higher leaf nutrient contents compared to non-inoculated controls, particularly an elevated K^+^/Na^+^ ratio, which was closely associated with the upregulated expression of Na^+^ extrusion-related genes. A substantial increase in proline content and the corresponding biosynthesis-related gene expression indicated that enhanced osmoprotectant synthesis played a critical role in GD17-conferred salt tolerance. Phytohormone levels and their signaling-related gene expression were also significantly upregulated in GD17-inoculated plants under salt stress. Moreover, transcription factor gene expression was markedly increased in GD17-treated plants following salt exposure. GD17 inoculation alleviated salt-induced photosynthetic inhibition, as demonstrated by improved photosynthetic efficiency and reduced suppression of photosynthesis-related gene expression. Transcriptional profiling further revealed that starch degradation, photorespiration, and the pentose phosphate pathway were crucial for GD17-mediated salt tolerance. Reduced oxidative damage, driven by enhanced antioxidative activity, further contributed to the observed protective mechanisms. This study demonstrates that the application of *Paraburkholderia* sp. GD17 concurrently enhances cucumber growth and salinity tolerance, effectively resolving the trade-off between growth and defense. Multi-level analyses provided comprehensive mechanistic insights into these synergistic effects.

## 1. Introduction

Soil salinity represents a critical challenge to global agricultural productivity, exacerbated by increasing NaCl accumulation and soil salinization [1]. Salinity imposes dual stresses on plants: osmotic stress, which limits water availability and suppresses shoot growth during early exposure, and ionic stress, which disrupts metabolic processes—particularly photosynthesis—through the toxic accumulation of Na^+^ and Cl^−^ in mature tissues [2]. Plants adapt to osmotic stress through mechanisms such as hydrotropic root growth, stomatal closure, and osmolyte accumulation. Meanwhile, tolerance to ionic stress relies on maintaining Na^+^ homeostasis via pathways like SOS-mediated exclusion. In Arabidopsis, the SOS3-SOS2 kinase complex phosphorylates SOS1, a plasma membrane-localized Na^+^/H^+^ antiporter, enabling Na^+^ extrusion under salt stress [3]. Cucumber, a globally important vegetable crop highly susceptible to salinity [4], exhibits analogous SOS-dependent regulation of Na^+^ homeostasis [5]. However, the potential of plant growth-promoting rhizobacteria (PGPR) to enhance these mechanisms remains largely unexplored.

The growth-defense trade-off paradigm underscores the necessity for precise hormonal coordination between stress adaptation and biomass production. Stress hormones such as abscisic acid (ABA) and salicylic acid (SA) antagonize growth-promoting hormones, including auxins (IAA), cytokinins (CKs), and gibberellins (GAs), forming a regulatory nexus that balances salt tolerance with developmental progression [6]. PGPR-mediated salinity tolerance often correlates with ABA signaling modulation, influencing stomatal dynamics, osmolyte biosynthesis, and ROS detoxification [7]. Despite this, comprehensive analyses of PGPR effects on hormonal crosstalk and downstream transcriptional networks remain limited.

Photosynthetic inhibition under salinity arises from multifaceted interactions, including stomatal closure limiting CO_2_ assimilation, enhanced photorespiration due to RuBisCo oxygenase activity, and ROS generation [8]. Ion toxicity further disrupts photosystem I/II (PSI/II) stability and electron transport efficiency [9]. Although PGPR inoculation alleviates photosynthetic inhibition through mechanisms yet to be fully elucidated [10], the interplay between bacterial priming and the transcriptional regulation of photoprotective pathways requires deeper investigation. Concurrently, ROS homeostasis—modulated by antioxidative enzymes and redox-sensitive signaling cascades—represents a key component of PGPR-conferred stress resilience [11,12].

While our previous work demonstrated the salt-tolerance efficacy of *Paraburkholderia* sp. GD17, a PGPR strain, in rice as a model monocotyledonous plant [13], transferring this beneficial trait to dicotyledonous crops such as cucumber still represents a critical agronomic challenge. Therefore, this study investigates the multifaceted mechanisms underlying salt tolerance enhancement in cucumber seedlings inoculated with *Paraburkholderia* sp. GD17. We hypothesize that GD17 orchestrates synergistic improvements in ionic homeostasis (via SOS pathway activation), hormonal balance, photosynthetic efficiency, and antioxidative defense, thereby resolving growth-defense trade-offs. Through integrated physiological, biochemical, and transcriptional analyses, we aim to (1) quantify GD17-mediated Na^+^/K^+^ redistribution and SOS-related gene expression; (2) elucidate phytohormone signaling dynamics and their transcriptional networks; (3) characterize photosynthetic adaptation mechanisms involving starch degradation, photorespiration, and pentose phosphate pathways; and (4) delineate ROS detoxification strategies. This multi-layered mechanism goes beyond our previous rice study, which primarily focused on ionic regulation, osmotic adjustment, and antioxidative defense [13]. These findings provide insights into optimizing PGPR application strategies for sustainable salinity management in horticultural crops.

## 2. Results

### 2.1. Salt Tolerance and Root Colonization Capability of the GD17 Strain

**Figure 1:**
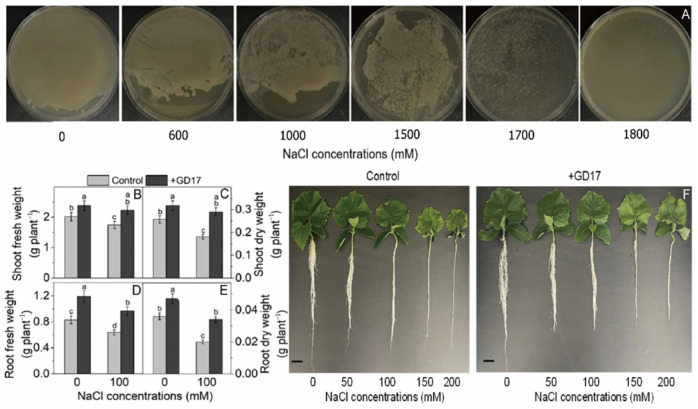
Effect of GD17 inoculation and/or salt stress on LB agar after 72-h incubation (**A**) and plant growth (**B**–**F**). Fresh and dry weights of shoots (**B**,**C**) and roots (**D**,**E**), along with representative photographs (**F**). Twenty-one-day-old plants were evaluated. The data were collected from three replicated experiments (*n* = 3), with 30 plants used in each experimental batch. Results are presented as means ± SD. Bars labeled with different lowercase letters indicate significant differences at *p* < 0.05.

GD17 grew well in LB medium containing 0–1800 mM NaCl and exhibited remarkable salt tolerance under high salinity conditions. Colony formation was progressively inhibited as NaCl concentration increased over the 3-day incubation period. However, significant growth was still observed at up to 1700 mM NaCl (Figure 1A). This robust halotolerance underscores GD17’s potential as a valuable microbial resource for improving plant resilience under saline conditions, which is of critical importance given the growing global challenge of soil salinization in agriculture.

Within 24 h post-inoculation (1 DPI), GD17 successfully colonized cucumber roots, reaching peak colonization density by 5 DPI (Figure S1A). Under salt stress, the bacterial population demonstrated stable colonization over a 7-day period, as evidenced by consistent CFU counts (Figure S1B). These results confirm GD17’s ability to establish sustained symbiotic relationships with host plants and exhibit resilience under saline conditions. When exposed to 100 mM NaCl for 7 days, GD17-inoculated plants exhibited significantly reduced biomass loss compared to non-inoculated controls: leaf fresh/dry weight reductions were 6.7%/8.5% versus 13%/29.6%, and root fresh/dry weight reductions were 18.5%/27.7% versus 23.3%/44% (Figure 1B–E). Collectively, these findings highlight GD17’s dual role in enhancing plant salt tolerance and promoting growth under both stressed and normal conditions (Figure 1B–F and Figure S1).

### 2.2. Levels of Na^+^ and Other Mineral Elements, and the Expression of Genes Involved in K^+^/Na^+^ Homeostasis

**Figure 2:**
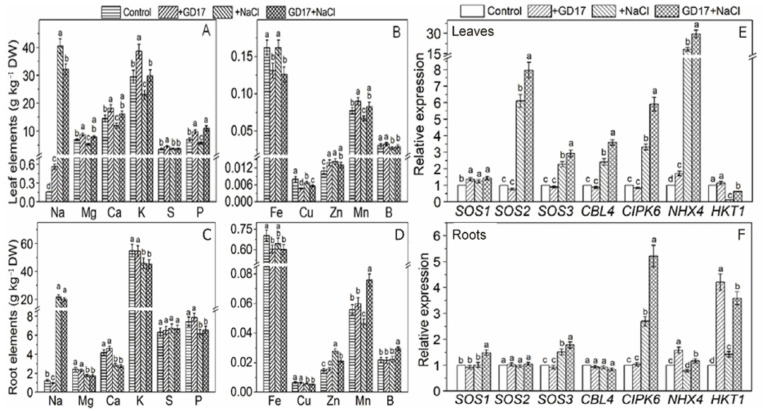
Effect of GD17 inoculation and/or salt stress on mineral element contents in plant leaves (**A**,**B**) and roots (**C**,**D**), and the expression of salt tolerance-related genes in plant leaves (**E**) and roots (**F**). Data are presented as means ± SD from three independent experiments (*n* = 3), with 20 seedlings per experiment. Different lowercase letters indicate significant differences at *p* < 0.05. The white line represents a break in the y-axis for clarity of data presentation.

Under salt stress, GD17 inoculation significantly improved ion homeostasis in cucumber leaves by reducing Na^+^ content by 19% and increasing K^+^ levels by 30%, leading to a 63% higher K^+^/Na^+^ ratio (0.93 vs. 0.57 in non-inoculated plants; Figure 2A). Additionally, GD17 enhanced the concentrations of Mg, Ca, P, and Mn in leaves while maintaining stable levels of S, Zn, and B. Notably, Fe and Cu concentrations moderately decreased in GD17-inoculated leaves (Figure 2A,B). In roots, GD17 induced distinct changes, specifically increasing Mn and B levels while decreasing Zn levels, with no significant effects on Na^+^, macronutrients (K, Mg, Ca, S, P), or Fe/Cu compared to controls (Figure 2C,D and Figure S2). These results indicate that GD17 selectively regulates mineral distribution across plant tissues, potentially via targeted nutrient partitioning mechanisms that enhance salt stress tolerance.

Under salt stress, GD17 inoculation elicited tissue-specific transcriptional responses in genes associated with salt tolerance (Figure 2E,F). *SOS1* expression was moderately upregulated, whereas GD17-treated leaves exhibited significantly stronger induction of *SOS2* (8-fold vs. 6-fold in non-inoculated plants) and *SOS3* (3-fold vs. 2-fold). Additionally, GD17 enhanced the expression of other key genes, including *CBL4* (3-fold), CIPK6 (6-fold), and *NHX4* (30-fold). Notably, *HKT1* expression decreased by 37% in GD17-inoculated leaves compared to an 88% reduction in controls (Figure 2E). In roots, the response patterns differed markedly, with only *CIPK6* and *HKT1* showing substantial GD17-mediated upregulation, while *SOS2* and *CBL4* remained unchanged (Figure 2F). Collectively, these findings highlight GD17’s role in modulating tissue-specific gene expression networks under salt stress.

### 2.3. Levels of Soluble Substances, Expression Profiles of Associated Genes, and Relative Water Content

**Figure 3:**
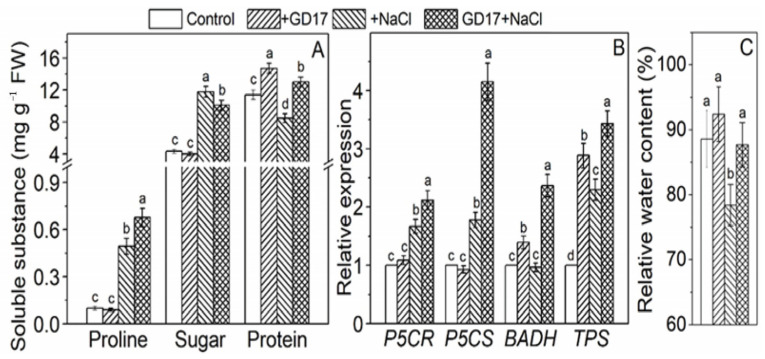
Effect of GD17 inoculation and/or salt stress on compatible solute contents (**A**), the expression of genes related to osmoregulatory substance synthesis (**B**), and relative water content (**C**) in plant leaves. Data are presented as means ± SD from three independent experiments (*n* = 3), with 20 seedlings per experiment. Different lowercase letters indicate significant differences at *p* < 0.05. The white line represents a break in the y-axis for clarity of data presentation.

GD17 inoculation significantly enhanced osmoprotectant accumulation under salt stress. Specifically, proline levels in GD17-treated leaves increased 6-fold compared to a 4-fold increase in non-inoculated controls. Soluble protein content rose by 14% in inoculated plants but decreased by 25% in controls. Conversely, soluble sugar levels exhibited a greater increase in non-inoculated plants (171%) compared to GD17-inoculated plants (133%) (Figure 3A). Under normal conditions, GD17 specifically increased soluble protein content by 29% without altering proline or sugar levels. These physiological responses were closely associated with the transcriptional regulation of key biosynthesis genes. Salt-stressed GD17 plants demonstrated a 2.0-fold induction of *P5CR* and a 4.2-fold induction of *P5CS*, surpassing the control levels (1.8-fold for both genes). *BADH* and *TPS* consistently exhibited higher expression levels in GD17-inoculated plants across all treatment conditions (Figure 3B). This enhanced capacity for osmoprotectant synthesis contributed to maintaining leaf water status, as evidenced by stable relative water content (RWC) in GD17 plants compared to a significant reduction (14%) in stressed controls (Figure 3C).

### 2.4. Levels of Phytohormones and Expression Profiles of Associated Genes in Signaling Pathways

**Figure 4:**
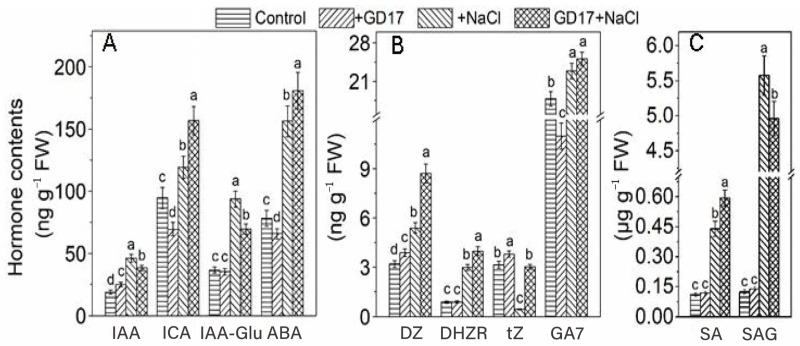
Effect of GD17 inoculation and/or salt stress on phytohormone contents and their derivatives in plant leaves. (**A**) Indole-3-acetic acid (IAA), indole-3-carboxylic acid (ICA), IAA-glutamic acid conjugate (IAA-Glu), and abscisic acid (ABA). (**B**) Dihydrozeatin (DZ), dihydrozeatin riboside (DHZR), trans-zeatin (tZ), and gibberellin (GA7). (**C**) Salicylic acid (SA) and its 2-*O*-β-D-glucoside (SAG). Data are presented as means ± SD from three independent experiments (*n* = 3), with 20 seedlings per experiment. Different lowercase letters indicate significant differences at *p* < 0.05. The white line represents a break in the y-axis for clarity of data presentation.

GD17 root inoculation differentially modulated leaf phytohormone profiles under salt stress. Specifically, GD17-inoculated plants exhibited a 100% increase in IAA, a 63% increase in ICA, and an 87% increase in IAA-Glu compared to increases of 130%, 26%, and 153%, respectively, in non-inoculated controls (Figure 4A). Under salt stress, ABA levels increased by 118% in GD17 plants, significantly higher than the 73% increase observed in controls. In contrast, GD17 inoculation reduced ABA levels by 33% under normal conditions. The accumulation of DZ and DHZR increased by 172% and 352%, respectively, in GD17 plants under stress, surpassing the control levels of 69% and 240%, respectively. Notably, tZ levels remained stable in GD17 plants but decreased to 14% of baseline levels in controls under salinity stress (Figure 4B). Salt stress elicited comparable GA7 accumulation in both GD17-inoculated and non-inoculated plants. However, under normal conditions, GD17 inoculation markedly decreased GA7 levels (Figure 4B). Under salt stress, SA content increased 4-fold in GD17 plants compared to a 3-fold increase in controls. Additionally, SA 2-*O*-β-D-glucoside (SAG) levels showed dramatic increases of 41-fold and 46-fold in GD17-inoculated and non-inoculated plants, respectively (Figure 4C).

Analysis of phytohormone-related gene expression revealed distinct regulatory patterns under salt stress. In leaves, *LAX* (IAA influx carrier) exhibited 140-fold higher expression in GD17-inoculated plants compared to 90-fold in controls relative to basal levels. *PIN* (IAA efflux carrier), however, showed a contrasting pattern with 18-fold and 60-fold increases in GD17 and control plants, respectively (Figure S3B). Root expression profiles largely mirrored leaf responses, except for *PIN*, which demonstrated GD17-specific enhancement (Figure S3D). Salt stress maximally activated ABA-related genes in GD17 leaves, with *PYL8* (3-fold), *ABF5* (4.5-fold), and *NCED2* (60-fold) showing significantly higher induction than in controls (Figure S3A,B). *NCED2* leaf expression correlated closely with ABA accumulation patterns (Figure 4A vs. Figure S3B). In roots, *NCED2* expression diverged sharply under stress, decreasing by 70% in GD17 plants and 90% in controls relative to basal levels. Constitutive downregulation of *NCED2* was observed in unstressed GD17 roots (Figure S3D). The expression of GA-related genes, *GID1* (encoding a GA receptor) and *RGL2* (encoding a DELLA protein, a negative regulator of GA signaling), exhibited salt-induced upregulation in leaves. Specifically, in GD17-inoculated plants, their expression levels reached 4-fold and 90-fold of the respective basal levels, respectively, compared to 2-fold and 68-fold increases in non-inoculated plants (Figure S3A,B). Similarly, their expression in roots generally followed the same trend as observed in leaves (Figure S3D). The expression of salicylic acid carboxyl methyltransferase (*SAMT*), an enzyme responsible for catalyzing the conversion of salicylic acid to its volatile methyl ester, demonstrated pronounced tissue-specific responses to salt stress (Figure S3B). In leaves, *SAMT* transcript levels increased by 1500-fold (GD17-inoculated) and 1000-fold (non-inoculated) relative to baseline measurements under salt treatment. This significant upregulation contrasted markedly with root responses, where salt stress induced only a 2-fold increase in *SAMT* expression. Notably, GD17 inoculation enhanced *SAMT* expression in roots even under normal conditions, resulting in a 4-fold increase compared to basal levels (Figure S3D). Salt stress induced significant upregulation of GA-related genes in leaves, with *GID1* (GA receptor) and *RGL2* (GA signaling repressor) showing 4-fold and 90-fold increases in GD17 plants compared to 2-fold and 68-fold in controls, respectively (Figure S3A,B). Root expression patterns were consistent with those in leaves, except for a less pronounced response of *RGL2* (Figure S3D). *SAMT* (SA methyltransferase) demonstrated striking tissue-specific regulation, with 1500-fold induction in GD17 leaves versus 1000-fold in controls under salinity, contrasting with only a 2-fold increase in roots (Figure S3C). Notably, *SAMT* expression was constitutively elevated by 4-fold in unstressed GD17 roots (Figure S3D).

### 2.5. Expression of Transcription Factor-Related Genes

**Figure 5:**
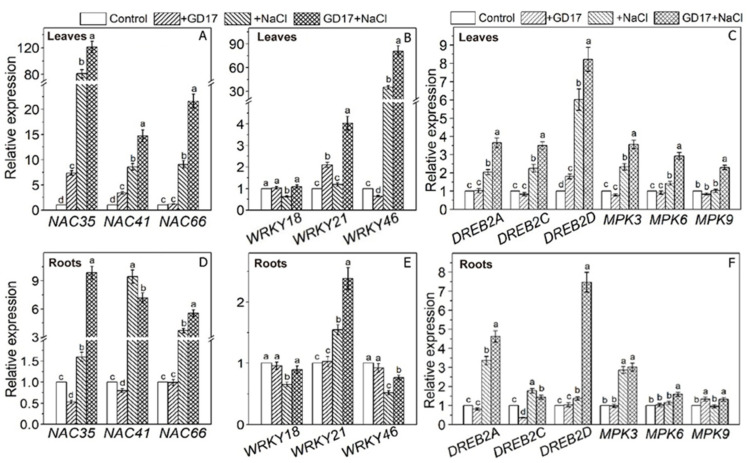
Effect of GD17 inoculation and/or salt stress on the expression of transcription factor-related genes in plant leaves (**A**–**C**) and roots (**D**–**F**). Data are presented as means ± SD from three independent experiments (*n* = 3), with 20 seedlings per experiment. Different lowercase letters indicate significant differences at *p* < 0.05. The white line represents a break in the y-axis for clarity of data presentation.

The expression of all three *NAC* genes (*NAC35*, -*41*, and -*66*) analyzed in this study uniformly exhibited a strong salt-induced upregulation in leaves, with a greater extent observed in GD17-inoculated plants compared to non-inoculated ones. Specifically, in GD17-inoculated plants, their expression levels reached 120-fold, 15-fold, and 21-fold of their respective basal levels, significantly higher than the increases of 80-fold, 7-fold, and 9-fold observed in non-inoculated plants, respectively (Figure 5A). The salt-induced upregulation of these genes was also evident in roots (Figure 5D). Three *WRKY* genes presented distinct expression patterns. Under normal conditions, GD17 inoculation did not influence the expression of *WRKY18* in either leaves or roots. However, under salt stress, it effectively reversed the inhibition of this gene’s expression (Figure 5B,E). The expression of *WRKY21* was upregulated by salt stress only in GD17-inoculated leaves but not in non-inoculated ones. In roots, however, its expression was elevated by salt stress in both types of plants (Figure 5B,E). The expression of *WRKY46* was dramatically upregulated by salt stress, with a greater extent observed in inoculated plants, showing an 80-fold increase compared to a 30-fold increase in non-inoculated plants. In contrast, its expression in roots was substantially downregulated in both types of plants (Figure 5B,E). The expression of three *DREB* genes (*DREB2A*, -*2C*, and -*2D*) generally exhibited a greater degree of salt-induced upregulation in GD17-inoculated plants. Notably, *DREB2D* expression reached more than 8-fold and 7-fold of their basal levels in the leaf and root, respectively (Figure 5C,F). GD17 inoculation did not affect the expression of *MPK3*, -*6*, and -*9* in leaves under normal conditions but significantly upregulated their expression in response to salt stress (Figure 5C). A similar trend was observed in roots (Figure 5F).

### 2.6. Chlorophyll Content and Photosynthetic Efficiency

**Figure 6:**
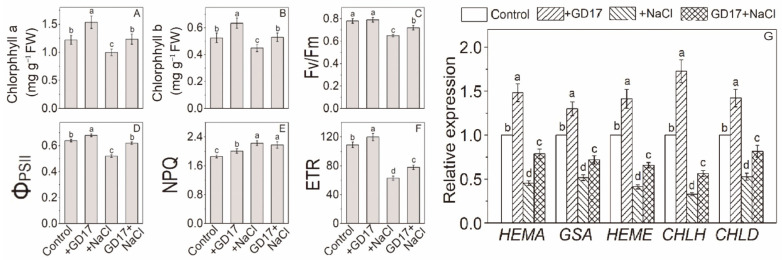
The effects of GD17 inoculation and/or salt stress on the chlorophyll pathway. (**A**) Chlorophyll a, (**B**) Chlorophyll b, (**C**) Maximum quantum efficiency of PSII photochemistry (Fv/Fm), (**D**) Actual quantum efficiency of PSII (ΦPSII), (**E**) Non-photochemical quenching of fluorescence (NPQ), (**F**) Linear electron transport rate (ETR), (**G**) Effect of GD17 inoculation and/or salt stress on the expression of chlorophyll biosynthesis-related genes. Data are presented as means ± SD from three independent experiments (*n* = 3), with 10 seedlings per experiment. Different lowercase letters indicate significant differences at *p* < 0.05.

The influence of salinity on photosynthetic pigments and photosynthetic efficiency was assessed by measuring chlorophyll (Chla and Chlb) content and chlorophyll fluorescence (ChlF) parameters after 7 consecutive days of NaCl exposure. Compared to their basal levels, Chla and Chlb decreased by approximately 18% and 15%, respectively, in non-inoculated plants under salt stress, but remained stable in GD17-inoculated plants (Figure 6A,B). Additionally, GD17 inoculation increased Chla and Chlb by 26% and 21%, respectively, under normal conditions. Under salt stress, non-inoculated plants exhibited a reduction in the ChlF parameters Fv/Fm, ΦPSII, and ETR by approximately 17%, 19%, and 42%, respectively, while NPQ increased by around 20%. Conversely, inoculated plants showed a smaller decrease in Fv/Fm (8%), ΦPSII (3%), and ETR (28%), with a modest increase in NPQ of about 9%, relative to their respective basal levels (Figure 6C–F). Under normal conditions, GD17-inoculated plants had higher values of ΦPSII, NPQ, and ETR than non-inoculated ones, with increases of approximately 6%, 4%, and 9%, respectively. Fv/Fm was comparable between the two groups (Figure 6C–F). Chlorophyll fluorescence imaging also revealed heterogeneity in salt-induced photosynthetic impairment within the leaf (Figure S4).

### 2.7. Expression of Photosynthesis-Related Genes

Under normal conditions, GD17 inoculation moderately enhanced the expression of chlorophyll biosynthesis-related genes, including *HEMA*, *GSA*, *HEME*, *CHLH*, and *CHLD*. However, under salt stress, these genes were significantly downregulated in non-inoculated plants by 55%, 48%, 59%, 67%, and 47%, respectively, compared to their respective basal levels. In contrast, in GD7-inoculated plants, the downregulation was much less pronounced, with reductions of only 21%, 28%, 34%, 44%, and 18% (Figure 6G). Similarly, salt stress markedly inhibited the expression of genes associated with the structure and function of PSI and PSII, as well as electron transport and photophosphorylation, to a greater extent in non-inoculated plants than in inoculated ones (Figures S5–S7). Under normal conditions, the expression of these genes was largely comparable between the two groups, with minor exceptions. Notably, *PsaF*, *PsbR*, *PsbY*, *PETE*, and *ATPγ* exhibited higher expression levels in inoculated plants (Figures S5–S7).

### 2.8. Expression of Genes Related to the Calvin Cycle and Starch Degradation

**Figure 7:**
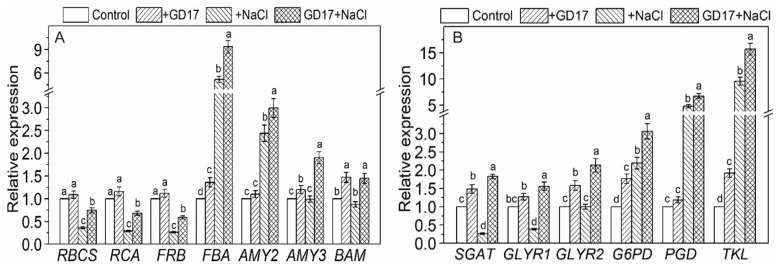
Effect of GD17 inoculation and/or salt stress on the expression of genes related to (**A**) Calvin cycle and starch degradation, (**B**) the photorespiration and the pentose phosphate pathway. Data are presented as means ± SD from three independent experiments (*n* = 3), with 20 seedlings per experiment. Different lowercase letters indicate significant differences at *p* < 0.05. The white line represents a break in the y-axis for clarity of data presentation.

Both *RBCS* and *RCA*, which encode the small subunit and activase of ribulose-1,5-bisphosphate carboxylase/oxygenase (RuBisCo), respectively, were significantly downregulated under salt stress in non-inoculated plants. Specifically, their expression levels decreased by approximately 64% and 71%, respectively, compared to reductions of 25% and 32% in inoculated plants, relative to basal levels. Under normal conditions, GD17 inoculation did not significantly alter their expression (Figure 7A). A similar pattern was observed for *FRB*, encoding fructose-1,6-bisphosphatase, a key enzyme in the Calvin cycle (Figure 7A). Conversely, *FBA*, encoding fructose-bisphosphate aldolase, exhibited salt-induced upregulation, particularly in inoculated plants, where it increased 8-fold compared to a 5-fold increase in non-inoculated plants (Figure 7A). Under salt stress, *AMY2* (α-amylase) expression increased more than 3-fold in inoculated lants and 2-fold in non-inoculated plants, while *AMY3* expression was elevated exclusively in inoculated plants (Figure 7A). Additionally, *BAM1* (β-amylase) expression was unaffected by salt stress but was moderately enhanced by GD17 inoculation (Figure 7A).

### 2.9. Expression of Genes Related to Photorespiration and Pentose Phosphate Pathway

In plants, toxic glyoxylate is primarily produced through the photorespiration pathway and is largely detoxified by serine/glyoxylate aminotransferase (SGAT) and glutamate/glyoxylate aminotransferase (GGAT), which convert it into glycine. Any residual glyoxylate can be further detoxified by glyoxylate reductase (GLYR). Under salt stress, *SGAT* expression was significantly downregulated by 73% in non-inoculated plants but upregulated by 82% in inoculated ones, relative to their respective basal levels. Additionally, under normal conditions, GD17 inoculation increased *SGAT* expression by 49% (Figure 7B). Similarly, *GLYR1* expression was downregulated by 62% in non-inoculated plants but upregulated by 56% in inoculated ones under salt stress. Under normal conditions, *GLYR1* expression was also enhanced by 27% in inoculated plants (Figure 7B). *GLYR2* expression remained unchanged in non-inoculated plants but was significantly upregulated in inoculated ones under salt stress (Figure 7B). Key genes in the pentose phosphate pathway, including *G6PD*, *6PGD*, and *TKL*, exhibited uniform salt-induced upregulation. Specifically, in inoculated plants, this upregulation reached 3-fold, 7-fold, and 16-fold of their basal levels, respectively, compared to approximately 2-fold, 5-fold, and 9-fold in non-inoculated ones (Figure 7B).

### 2.10. Antioxidation and Oxidative Damage

**Figure 8:**
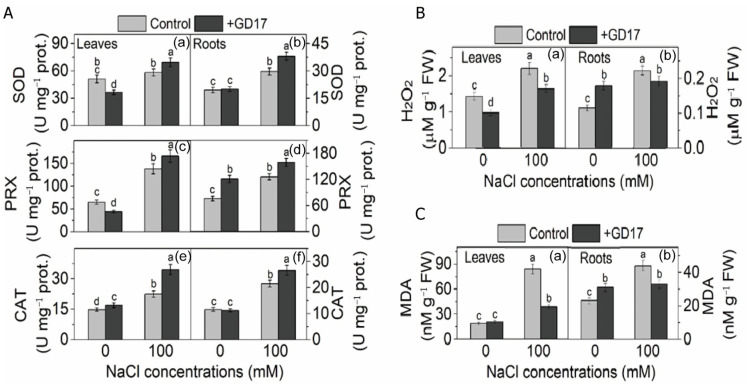
Effects of GD17 inoculation and/or salt stress on the antioxidant system of plants. (**A**) antioxidative enzymes, (**B**) peroxide hydrogen (H_2_O_2_), and (**C**) malondialdehyde (MDA) production in plant leaves (**Aa**,**Ac**,**Ae**,**Ba**,**Ca**) and roots (**Ab**,**Ad**,**Af**,**Bb**,**Cb**). Data are presented as means ± SD from three independent experiments (*n* = 3), with 20 seedlings per experiment. Different lowercase letters indicate significant differences at *p* < 0.05.

Exposure to salinity significantly enhanced SOD activity in GD7-inoculated plants by 36% in leaves and 95% in roots, compared to increases of 12% in leaves and 52% in roots of non-inoculated plants, relative to their respective basal levels. Under normal conditions, however, GD17 inoculation reduced SOD activity in leaves but had no effect on roots (Figure 8(Aa,Ab)). Similarly, under salt stress, PRX activity was markedly elevated in GD17-inoculated plants, with increases of 155% in leaves and 110% in roots, compared to 112% in leaves and 66% in roots of non-inoculated plants (Figure 8(Ac,Ad)). Additionally, CAT activity increased by 133% in inoculated leaves and 129% in inoculated roots, significantly higher than the 51% and 85% increases observed in non-inoculated plants (Figure 8(Ae,Af)). Under normal conditions, PRX activity decreased by 32% in GD17-inoculated leaves. However, it increased by 60% in roots (Figure 8(Ac,Ad)), while CAT activity was slightly enhanced in inoculated leaves but remained unchanged in roots (Figure 8(Ae,Af)). Furthermore, under salt stress, H_2_O_2_ content increased by 54% in leaves and 93% in roots of non-inoculated plants, while MDA levels rose by 342% in leaves and 91% in roots. These increases were significantly higher than those observed in inoculated plants, where H_2_O_2_ increased by 15% in leaves and 67% in roots, and MDA by 105% in leaves and 44% in roots, relative to their respective basal levels (Figure 8(Ba,Bb)). Under normal conditions, GD17 inoculation reduced H_2_O_2_ content by 32% in leaves but increased it by 56% in roots, while it had minimal impact on MDA levels in leaves but elevated them by 35% in roots (Figure 8(Ca,Cb)).

### 2.11. Expression of Antioxidase-Related Genes

Under salt stress, the expression of *CSD* (Cu-Zn SOD) and *MSD* (Mn-SOD) in inoculated leaves increased by 187% and 118%, respectively, significantly exceeding the 68% and 64% increases observed in non-inoculated plants. Conversely, *FSD* (Fe-SOD) expression increased by 59% in inoculated leaves but decreased by 25% in non-inoculated leaves relative to basal levels (Figure S8A). Under normal conditions, GD17 inoculation induced a 100% increase in *FSD* leaf expression while having minimal effects on *CSD* and *MSD* (Figure S8A). In roots, salt stress upregulated *CSD* expression to 3.8-fold in inoculated plants and 2.7-fold in non-inoculated plants (Figure S8B). Meanwhile, *FSD* expression was moderately upregulated, whereas *MSD* was downregulated in both plant groups regardless of treatment. Under salt stress, *PRX47* expression increased to 3.9-fold of baseline in inoculated leaves but decreased by 37% in non-inoculated leaves. GD17 inoculation elevated *PRX47* expression by 66% under normal conditions (Figure S8A). Root *PRX47* expression reached 15-fold and 8-fold of the basal levels in inoculated and non-inoculated plants under stress (Figure S8B). *PRX73* expression rose to 54-fold in inoculated leaves and 114-fold in non-inoculated leaves under salinity (Figure S8A). However, root *PRX73* showed insensitivity to salt stress, with only moderate GD17-induced upregulation irrespective of NaCl presence (Figure S8B). Salt stress significantly enhanced *CAT1* and *CAT3* expression in GD17-inoculated plants. Specifically, *CAT1* expression increased by 124% in leaves and nearly 10-fold in roots, while *CAT3* expression rose by 290% in leaves and 16-fold in roots. These increases markedly surpassed those observed in non-inoculated plants, where *CAT1* expression increased by 40% in leaves and 5-fold in roots, and *CAT3* expression increased by 58% in leaves and 4-fold in roots relative to their respective basal levels (Figure S8A,B).

## 3. Discussion

Plant-associated microbes such as Pseudomonas and Bacillus can enhance the crop’s stress resistance and increase yield by regulating plant hormones, improving nutrient absorption, and activating the plant’s immune system [14]. Different PGPR strains produce similar phenotypes, but the molecular mechanisms they exhibit may differ widely [15]. This study systematically elucidates the mechanism by which rhizobacterium GD17 enhances cucumber seedling salt tolerance through four synergistic strategies: restoring ionic/osmotic homeostasis, modulating phytohormone signaling, optimizing carbon metabolism, and activating antioxidant defenses, with potential crosstalk among these pathways. Given that soil microbiomes and soil-root interactions are absent in hydroponics, the observed effects may differ under natural field conditions.

GD17 inoculation effectively mitigated salt-induced ionic imbalance, particularly by preserving the K^+^/Na^+^ ratio—a critical determinant of salt tolerance [16]. This was achieved through coordinated regulation of Na^+^ transport systems: (1) Activation of the SOS pathway, evidenced by consistent upregulation of *SOS2* in leaves under salinity (Figure 2E), aligns with H_2_S-mediated salt tolerance mechanisms reported in previous studies [17]; (2) Restricted root-to-shoot Na^+^ translocation, as indicated by reduced leaf Na^+^ accumulation (Figure 2A,C), is consistent with endophyte-mediated salt tolerance strategies observed in other species [18]; (3) Enhanced expression of key Na^+^ transporters, including *CIPK6*, *CBL4*, and *NHX4* (Figure 2E), suggests complementary mechanisms for maintaining ion homeostasis. Notably, the strong induction of *CsNHX4* implicates its role in vacuolar Na^+^ sequestration, warranting further functional characterization despite limited prior reports. The dual regulation of *HKT1* presents an intriguing pattern: root-specific upregulation in GD17-inoculated plants versus leaf downregulation under salinity (Figure 2E,F). This tissue-specific modulation reconciles previous contradictory findings—while Arabidopsis *hkt1* mutants exhibit improved tolerance through cellular Na^+^ reduction [19], tomato *HKT1* enhances Na^+^ exclusion and K^+^ uptake [20]. Our results suggest that root-specific activation of *HKT1* contributes to GD17-conferred salt tolerance, corroborating the benefits of root *HKT1* overexpression observed in other species [21]. Additionally, proline plays dual roles as both a ROS scavenger and a stabilizer of macromolecules [22]. In this study, GD17-inoculated plants demonstrated significantly elevated proline levels under salt stress (Figure 3A), consistent with upregulated expression of proline synthesis genes (Figure 3B), suggesting enhanced capacity for osmotic adjustment in these plants.

GD17 inoculation modulated multiple phytohormone pathways. ABA signaling and MAPK cascades play pivotal roles in the regulation of osmolyte production. For instance, overexpression of the ABA receptor *PYL8* in Arabidopsis significantly enhances the expression of the proline synthase gene *P5CS*, thereby improving drought tolerance [23]. Abscisic acid (ABA), a key stress-responsive hormone, plays a central role in balancing plant growth and defense under osmotic stress. In the ABA signaling pathway, stress-induced increases in ABA levels lead to its binding with PYR/PYL receptors, which inactivate PP2C phosphatases and subsequently activate SnRK2 kinases. These kinases phosphorylate ion transporters and AREB/ABF transcription factors, initiating downstream responses [24]. GD17-inoculated plants exhibited markedly elevated ABA levels and upregulated expression of *PYL8* and *ABF5* under salt stress (Figure 4, Figure 5C and Figure S3), underscoring the importance of ABA signaling in GD17-mediated salt tolerance. In cucumber, 14 *MAPK* genes exhibit stress-modulated expression, with *MPK3*, *MPK6*, and *MPK9* showing enhanced activation in GD17-inoculated plants (Figure 5C,F). MAPKs interact with hormone pathways (ABA, SA, IAA, GA) and regulate ionic homeostasis, as demonstrated by the Arabidopsis AtMPK6-mediated phosphorylation of SOS1, which reduces Na^+^ accumulation [25]. In addition, *NAC35*, *NAC41*, and *NAC66* exhibit significant upregulation in GD17-inoculated plants under salt stress (Figure 5A,D). These genes are known to respond to ABA and salinity [26]. *DREB2A*, *DREB2C*, and *DREB2D* were significantly upregulated in GD17-inoculated plants (Figure 5C,F). This aligns with the functional divergence of the DREB subfamilies, and DREB2 members mediate responses to drought, salinity, and osmotic stress [27]. These findings are consistent with previous reports indicating that plant growth-promoting rhizobacteria (PGPR) enhance salt tolerance through multiple regulatory mechanisms, including phytohormone signaling (e.g., ABA), transcription factor regulation (e.g., DREB), and ROS/calcium signaling pathways [11]. Additionally, salt-stressed GD17-inoculated plants showed increased GA7 content and *GID1* expression (Figure 4B and Figure S3A). In contrast, non-inoculated plants exhibited significant upregulation of *RGL2*, a gene encoding DELLA proteins, which are known inhibitors of plant growth and development (Figure S3B). This suggests that GD17 mitigates the inhibitory effects of DELLA proteins on plant growth under salt stress by enhancing GID1-mediated DELLA degradation. Furthermore, GD17-inoculated plants displayed elevated IAA levels and increased expression of auxin transporters *LAX* and *PIN* (Figure 4A and Figure S3), facilitating root adaptation and ion uptake. Salicylic acid (SA) accumulation and dramatic upregulation of *SAMT* (1500-fold in GD17-inoculated leaves; Figure S3C) indicated systemic methylsalicylate (MeSA) signaling, potentially linked to SOS pathway activation [28].

Emerging evidence demonstrates a strong link between plant salt tolerance and photosynthetic capacity [9]. GD17 inoculation significantly improved PSII photochemical efficiency and alleviated salt-induced photochemical damage to the photosynthetic apparatus (Figure 6 and Figure S4). This protective mechanism likely contributes to GD17-mediated salt tolerance by ensuring adequate carbon and energy supplies for osmolyte synthesis while minimizing ROS overproduction through optimized photosynthetic electron transport [29]. Transcriptional regulation of chlorophyll (Chl) biosynthesis genes plays a pivotal role in determining Chl dynamics under salt stress (Figure 6). The moderate reduction in Chl levels observed in GD17-inoculated plants (Figure 6A,B) reflects an adaptive strategy that balances photoprotection via stabilization of the PS-LHC complex and minimizes ROS generation from free Chl intermediates [30]. This finding is consistent with PGPR-mediated stress adaptation mechanisms, where precise regulation of Chl content maintains optimal photosynthetic efficiency under adverse conditions [31]. Salt stress severely compromises the functionality of photosynthetic machinery, including components involved in light reactions (PSI/PSII reaction centers, oxygen-evolving complexes) and Calvin cycle enzymes (RuBisCO subunits, fructose-1,6-bisphosphatase). In non-inoculated plants, the expression of genes encoding these proteins was reduced by over 50% under salt stress (Figure 7A and Figures S5–S7). However, GD17 inoculation significantly mitigated this suppression. Notably, fructose-1,6-bisphosphate aldolase (FBA) displayed contrasting upregulation under salinity conditions (Figure 7A), consistent with prior findings on the stress-responsive expression of *CsFBA3* [32]. As a key enzyme with dual roles in the Calvin cycle and glycolysis/gluconeogenesis, FBA plays an essential role in osmolyte synthesis and enhancing plant resilience to salinity stress [33]. Its contribution to salinity tolerance has been validated through in vitro assays [34]. The pentose phosphate pathway (PPP) serves as a central metabolic hub, providing NADPH and essential intermediates for carbon/nitrogen metabolism and biosynthetic processes. Salt stress markedly upregulated the expression of glucose-6-phosphate dehydrogenase (*G6PD*) (Figure 7B), consistent with observations in soybean and other species [35,36]. Similarly, the salinity-responsive upregulation of 6-phosphogluconate dehydrogenase (*6PGD*) and transketolase (*TKL*), particularly in GD17-inoculated plants (Figure 7B), aligns with their established roles in stress adaptation through metabolic coordination [37,38]. Collectively, these findings suggest that the synergistic interplay between PPP-driven carbon assimilation and catabolism underpins GD17-enhanced salt tolerance.

Plants activate starch degradation under salt stress to produce osmoprotectants, a process mediated by α-amylases (AMY1/3) and β-amylases (BAM1). Heterologous overexpression of the sweet potato *BAM1* gene enhances salinity tolerance in Arabidopsis by promoting osmoprotectant synthesis and reducing ROS accumulation [13]. The ABA signaling module SnRK2-AREB/ABF regulates this process via transcriptional activation of *BAM1* and *AMY3* [39]. In GD17-inoculated plants, the coordinated upregulation of *AMY*/*BAM* expression (Figure 7A) and enhanced ABA signaling (Figure 4) suggest that ABA-mediated starch catabolism plays a critical role in salt tolerance, consistent with established PGPR mechanisms [40]. Salt stress-induced stomatal closure reduces CO_2_ availability at RuBisCO active sites, enhancing its oxygenase activity and initiating photorespiration [8]. While photorespiration mitigates photoinhibition by dissipating excess energy and recycling CO_2_ for photosynthesis, it generates toxic intermediates (2-phosphoglycolate, glycolate, glyoxylate) that require efficient detoxification. GD17 inoculation upregulated *SGAT* and *GLYR1* expression under salinity (Figure 7B), suggesting enhanced glyoxylate detoxification contributes to salt tolerance. This aligns with recent findings that photorespiratory enzyme serine hydroxymethyltransferase (SHMT) improves cucumber salinity resilience through antioxidant regulation and osmotic adjustment [41]. GD17 inoculation markedly mitigated salt-induced oxidative damage, as indicated by reduced malondialdehyde (MDA) accumulation compared to non-inoculated plants (Figure 8C). Enhanced activities of key ROS-detoxifying enzymes—superoxide dismutase (SOD), peroxidase (PRX), and catalase (CAT)—were observed in GD17-treated plants across tissues (Figure 8A), consistent with the upregulated expression of their corresponding genes (Figure S8A,B). This antioxidant activation was associated with elevated expression of *CsMPK3/6/9* (Figure 5C,F), supporting MAPK-mediated regulation of ROS-scavenging genes [42]. Furthermore, upregulation of transcription factor genes *NAC*, *WRKY*, and *ERF/DREB* (Figure 5) potentially coordinates antioxidant responses, corroborating previous findings [12], and aligning with PGPR-mediated stress adaptation mechanisms [11,43]. Collectively, these results demonstrate that GD17 enhances salt tolerance through ROS reduction and elevated antioxidant capacity.

## 4. Materials and Methods

### 4.1. Plant Material and Treatment

Cucumber seeds (*Cucumis sativus* L. cv. Zhongnong 26) were surface-sterilized by immersion in 75% ethanol for 2 min and subsequently germinated on moistened filter paper under dark conditions at 30 °C for 48 h. Uniformly germinated seedlings were transferred to a hydroponic system containing half-strength Hoagland nutrient solution and maintained under controlled environmental conditions: 28 °C/22 °C (day/night), 200 μmol m^−2^ s^−1^ light intensity, and a 14-h photoperiod with 10 h of darkness. Seven-day-old seedlings were inoculated with the GD17 strain via bacterial suspension (1 × 10^8^ CFU mL^−1^ in Hoagland solution), achieving a final concentration of approximately 1 × 10^6^ CFU mL^−1^. Salt stress treatments (0, 50, 100, 150, and 200 mM NaCl) were initiated 7 days post-inoculation. Based on preliminary observations (Figure 1F), subsequent analyses focused on plant growth parameters after 7 days of exposure to 100 mM NaCl and physiological/molecular responses assessed at 48 h post-treatment. Four treatment groups were established by applying the corresponding treatments to cucumber roots: Control, GD17, NaCl, and GD17 + NaCl. All experiments were conducted with three independent biological replicates.

### 4.2. Evaluation of GD17 Colonization Efficiency Within the Root Interior

The colonization efficiency of GD17 within cucumber roots was quantified by enumerating colony-forming units (CFU). Root samples were collected at 0, 1, 3, 5, and 7 days post-inoculation (dpi) and at 1, 3, 5, and 7 days post-salt exposure (dps). Surface sterilization was achieved using a 5% sodium hypochlorite solution for 10 min, followed by three rinses with sterile water. Sterilized root tissues were homogenized in 15 mL of 0.9% NaCl solution, agitated at 150 rpm for 30 min, and subsequently serially diluted. Aliquots were plated onto LB agar, and colonies were enumerated after incubation at 28 °C for 48 h.

### 4.3. Measurement of Plant Growth and Mineral Element Contents

Plant growth was evaluated by measuring fresh and dry weights after 7 days of salt exposure. Samples (*n* = 30 per group) were rinsed with deionized water, partitioned into shoots and roots, and individually weighed for fresh biomass determination. Subsequently, the tissues were oven-dried at 80 °C for 48 h to determine dry mass. For elemental profiling, approximately 0.1 g of dried tissue powder was digested with 5 mL of concentrated HNO_3_ and analyzed using ICP-AES (iCAP 6000 Series, Thermo Fisher Scientific, Waltham, MA, USA). The concentrations of Na, Mg, Ca, K, S, P, Fe, Cu, Zn, Mn, and B were quantified.

### 4.4. Quantification of Phytohormone Contents

Phytohormone contents were quantified from 100 mg of frozen leaf tissue (*n* = 20 per group) using UPLC-Orbitrap MS. Tissues were pulverized under liquid nitrogen and extracted with 1 mL of ice-cold 50% aqueous acetonitrile (ACN; *v*/*v*) through sequential sonication (3 min, 4 °C) and rotary extraction (120 rpm, 30 min). Following centrifugation (12,000× *g*, 10 min, 4 °C), supernatants were purified via C18 SPE cartridges (preconditioned with methanol and water) and dried under a gentle nitrogen stream. Final analyses were conducted on a Vanquish UPLC/Q Exactive Orbitrap MS system.

### 4.5. Determination of Antioxidant Enzyme Activity and Oxidative Stress Markers

Leaf and root tissues (0.2 g each) were harvested and pooled from 20 plants per treatment group, and ground into a fine powder under liquid nitrogen using a mortar and pestle. The powder was suspended in 5 mL of pre-chilled extraction buffer consisting of 50 mM PBS (pH 7.8), 0.1 mM EDTA, 1% (*v*/*v*) Triton X-100, and 4% (*w*/*v*) polyvinylpyrrolidone, followed by incubation on ice for 10 min. After centrifugation at 12,000× *g* and 4 °C for 15 min, the supernatant (crude enzyme extract) was collected for the determination of superoxide dismutase (SOD; EC 1.15.1.1), peroxidase (PRX; EC 1.11.1.7), and catalase (CAT; EC 1.11.1.6) activities using a UV-1800 spectrophotometer (Shimadzu, Kyoto, Japan). Enzyme unit definitions followed those described by Hao et al. [44]. Total protein content was quantified using the Bradford method [45]. For the assessment of oxidative stress markers, the powder was homogenized in 5 mL of 0.1% (*w*/*v*) trichloroacetic acid (TCA), incubated on ice for 10 min, and centrifuged at 12,000× *g* and 4 °C for 10 min. A 1 mL aliquot of the supernatant was mixed with 4 mL of reaction solution containing 0.5% (*w*/*v*) 2-thiobarbituric acid (TBA) dissolved in 20% (*w*/*v*) TCA. Malondialdehyde (MDA) and hydrogen peroxide (H_2_O_2_) levels were measured according to the methods described by Hao et al. [44].

### 4.6. Assessment of Photosynthesis-Related Parameters

Chlorophyll fluorescence parameters were analyzed using a fluorescence imaging system (FluorCam, Photon Systems Instruments, Brno, Czech Republic). Briefly, plants were dark-adapted for 15 min to determine the minimum fluorescence yield (F_0_), followed by exposure to a 1.0-s saturating light pulse (1500 μmol m^−2^ s^−1^) to record maximum fluorescence yield (F_m_). Subsequently, steady-state fluorescence yield (F_s_) was assessed under continuous actinic illumination (30 μmol·m^−2^·s^−1^) for 15 min. Key photosynthetic parameters, including the maximal photochemical efficiency of PSII (F_v_/F_m_ = (F_m_ − F_0_)/F_m_), actual photochemical efficiency of PSII (ΦPSII), electron transport rate (ETR), and non-photochemical quenching (NPQ), were calculated using the instrument’s integrated software. For chlorophyll quantification, total chlorophyll was extracted from leaf tissues using 80% (*v*/*v*) acetone, and absorbance measurements were performed according to the spectrophotometric protocol of Zhang and Qu [46].

### 4.7. Estimation of Compatible Solutes and Relative Water Content (RWC)

Fresh leaf samples (pooled from 20 plants per treatment group) were homogenized in liquid nitrogen and extracted with 3% (*w*/*v*) sulfosalicylic acid. Following centrifugation at 10,000× *g* and 4 °C for 10 min, the supernatant was mixed with an equal volume of ninhydrin reagent dissolved in cold glacial acetic acid, incubated in a water bath at 100 °C for 30 min, and rapidly cooled on ice. Proline concentration was quantified by measuring absorbance at 520 nm using a microplate reader (FLUOstar Omega, BMG Labtech, Ortenberg, BW, Germany). Soluble sugars were determined using the anthrone-sulfuric acid method [46], while soluble protein content was assayed according to the Bradford method [45]. For RWC determination, leaf disks (1.5 cm diameter) excised from the youngest fully expanded leaves were immediately weighed to obtain fresh weight (FW). The disks were subsequently hydrated in distilled water at 25 °C for 24 h to determine turgid weight (TW), followed by oven-drying at 80 °C for 48 h to record dry weight (DW). RWC was calculated as follows:

RWC (%) = [(FW − DW)/(TW − DW)] × 100

### 4.8. Analysis of Gene Expression by qRT-PCR

Gene expression analysis was conducted using quantitative reverse transcription PCR (qRT-PCR) on a LightCycler 96 system (Roche, Basel, Switzerland). Leaf and root tissues (pooled from 20 plants per treatment group) were homogenized in liquid nitrogen. Total RNA was isolated using the RNAiso Plus Kit (TaKaRa, Dalian, China), followed by first-strand cDNA synthesis with the PrimeScript RT Reagent Kit (TaKaRa) according to the manufacturer’s instructions. Amplification reactions were carried out following the thermocycling parameters described by Qu et al. [47]. Normalization was achieved using three reference genes (*Actin7*, *EF1α*, and *TUA*), and relative gene expression levels were calculated using the 2^−ΔΔCT^ method. Primer sequences are listed in Supplementary Table S1.

### 4.9. Statistical Analysis

Statistical analyses were conducted using SAS 9.4 software (SAS Institute, Cary, NC, USA). All data were obtained from three independent biological replicates (*n* = 3) and are presented as mean ± standard deviation (SD). Significant differences among treatments (*p* < 0.05) were evaluated by one-way ANOVA followed by Duncan’s multiple range test. Four treatment groups were compared globally, and different lowercase letters (a, b, c, d) indicated significant differences.

## 5. Conclusions

**Figure 9:**
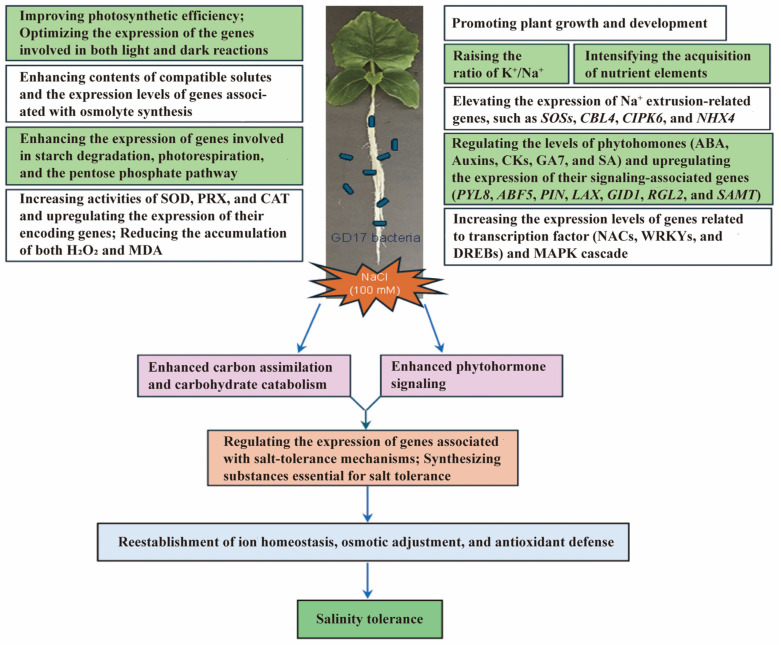
Mechanisms of GD17-mediated salt tolerance in cucumber seedlings.

This study provides the first evidence that root inoculation with *Paraburkholderia* sp. GD17 promotes cucumber growth under both normal and salt-stress conditions while enhancing salinity tolerance. The underlying mechanisms involve multiple processes, including: (1) regulation of ionic homeostasis, particularly optimization of the K^+^/Na^+^ ratio; (2) synthesis of osmolytes; (3) rewiring of phytohormone signaling pathways; (4) coordination of carbon assimilation and carbohydrate catabolism; (5) mitigation of oxidative stress through enhanced antioxidant defense systems; and (6) transcriptional reprogramming mediated by stress-responsive transcription factors and the MAPK signaling pathway. These processes function synergistically via cross-compartmental signaling networks. Notably, phytohormone signaling pathways and carbon metabolism networks serve as central regulatory hubs, orchestrating the expression of salt-responsive genes and providing essential carbon skeletons and metabolic energy for the synthesis of stress-mitigating compounds. This systemic reprogramming enables cucumber plants to achieve three key adaptive responses: restoration of ionic equilibrium, osmotic adjustment through compatible solute accumulation, and reinforcement of redox homeostasis via elevated antioxidant capacity. The integrative interplay of these mechanisms, as summarized in Figure 9, collectively enhances the salt tolerance of GD17-inoculated cucumber plants.

## Data Availability

The original contributions presented in this study are included in the article; further inquiries can be directed to the corresponding authors.

## Supplementary Materials

The following supporting information can be downloaded at: https://www.mdpi.com/article/10.3390/plants15071104/s1, Table S1: The specific primer sequences for the qRT-PCR; Figure S1: Colonization efficiency of GD17 inside roots as indicated by colony-forming units (CFU); Figure S2: Representative photographs illustrating the effect of GD17 inoculation on plant development; Figure S3: Effect of GD17 inoculation and/or salt stress on the expression of phytohormone synthesis and signaling-related genes in plant leaves and roots; Figure S4: Representative pictures of chlorophyll a fluorescence imaging; Figure S5: Effect of GD17 inoculation and/or salt stress on the expression of PSI-related genes; Figure S6: Effect of GD17 inoculation and/or salt stress on the expression of PSII-related genes; Figure S7: Effect of GD17 inoculation and/or salt stress on the expression of genes related to the electron transport and photophosphorylation; Figure S8: Effect of GD17 inoculation and/or salt stress on the expression of antioxidase-related genes in plant leaves and roots.

## Abbreviations

The following abbreviations are used in this manuscript:

PGPR	Plant growth-promoting rhizobacteria
ABA	Abscisic acid
SA	Salicylic acid
IAA	Indole-3-acetic acid
CKs	cytokinins
GAs	gibberellins
DPI	Day post-inoculation
RWC	Relative water content
ICA	indole-3-carboxylic acid
IAA-Glu	IAA-glutamic acid conjugate
DZ	Dihydrozeatin
DHZR	dihydrozeatin riboside
tZ	trans-zeatin
SAG	Salicylic acid 2-*O*-β-D-glucoside
H_2_O_2_	Peroxide hydrogen
MDA	Malondialdehyde
SOD	Superoxide dismutase
PRX	Peroxidase
CAT	Catalase
ROS	Reactive oxygen species
Fv/Fm	Maximum quantum efficiency of PSII photochemistry
ΦPSII	Actual quantum efficiency of PSII
ETR	Linear electron transport rate
NPQ	Non-photochemical quenching of fluorescence
