# Supplementary material for: Paraburkholderia Mediates Salt Stress Alleviation in Cucumber Seedlings
Source: Plants (Basel). 2026 Apr 3;15(7):1104. doi: 10.3390/plants15071104 (PMC13074637; doi:10.3390/plants15071104)
Supplement: Supplementary file 1 [file plants-15-01104-s001.zip › plants-4206777-supplementary.pdf]

Supplementary Table S1

The specific primer sequences for the qRT-PCR

Gene ID	Gene symbol	Function	Forward primer (5'–3')<br>Reverse primer (5'–3')	Amplified length (bp)
CsaV3_6G041900	*ACT7*	Actin (internal control)	TGAACTGAGATTGGTTGGCGT<br>TTGCCCAAATCTGGAGGGTC	178
CsaV3_6G021670	*EF1α*	Elongation factor 1-alpha (internal control)	CAGACAAGCCACTCCGTCTT<br>GCCTCGGGTAGAGATTCGTG	181
CsaV3_4G000060	*TUA*	Tubulin alpha chain (internal control)	CTCCCTCCTTTTGGAGCGTT<br>GAAGCACAGCAACGTCAGTG	161
CsaV3_7G008500	*ABF5*	ABA responsive element binding factor	ATCACGGGCTCGTAAACAGG<br>CATGGGCCAGTCAGTGTTCT	192
CsaV3_4G037030	*AMY2*	Alpha-amylase	GATGGGATGGTGGCCTTCAA<br>TGCACTGAGTCACCCCATTC	180
CsaV3_5G032400	*AMY3*	Alpha-amylase	CGATTGCCCTCGATCCTCTC<br>AGCTCTTTCGGCCATGACAA	138
CsaV3_6G002740	*ATPδ*	Delta subunit of ATP synthase	TCTACAGCCGCATTCAGCAA<br>TGCACCTGTTTCGCGATTTG	186
CsaV3_2G032160	*ATPβ*	Beta subunit of ATP synthase	GATCGAAAAGGCCGCTCTCT<br>AATGGCGGTGTCTCTTTCGT	139
CsaV3_1G010750	*ATPγ*	Gamma subunit of ATP synthase	TCCCAGAATTCAACCCCTGC<br>CTCTACGGACTTTGGCAGCA	148
CsaV3_1G001830	*BADH*	Betaine aldehyde dehydrogenase	ACGTGCCAAGTATTTGCGTG<br>TCCCATGCAGCTTCTTCCAG	111
CsaV3_3G030250	*BAM1*	Beta-amylase 1	AATGTCGCAGTCCTTCAGGG<br>CGAACTTTGAGGCCAAACCG	159
CsaV3_4G037250	*CAT1*	Catalase	CCGAGAGGTATCCTCACCCA<br>AAATGCTTGGCCTCACGTTG	270
CsaV3_6G031490	*CAT3*	Catalase	CACTGGAAACCAACTTGCGG<br>ATCGTCGTATCCAACGGCTC	200
CsaV3_3G019930	*CBL4*	Calcineurin B-like protein	GAGCGAGGTAGAGGCATTGT<br>CCATTGCGCTTCACGTCAAAT	168
CsaV3_6G039120	*CHLD*	Mg-chelatase subunit D	CCTTCAGCACAAGAATTGAAGGT<br>CCTTGGCAAAGCCAGTTGAC	131
CsaV3_4G011730	*CHLH*	Mg-chelatase subunit H	TGTCGTCCTTGGTGTCATCG<br>GACGAACTTCGGGACTGGTT	201
CsaV3_2G003670	*CIPK6*	CBL-interacting serine/threonine-protein kinase	GGTGGTGGAGGTGAAGAAGG<br>CCGTCGGAGAAGTCCAAACA	107
CsaV3_1G039510	*CSD*	Cu-Zn superoxide dismutase	GCCACATTTCAACCCTGCTG<br>GTCCACCCTTGCCAAGATCA	209
CsaV3_4G026790	*DREB2A*	Dehydration-responsive element-binding protein	ATGGCTTGGCACTTTCTCCA<br>ACTTTCACCTTCAGTTCCTCCA	285
CsaV3_6G002010	*DREB2C*	Dehydration-responsive element-binding protein	GGAGTAGGCTTTGGCTTGGT<br>GTGAACTCCTCAGGCACACA	259

CsaV3_2G029160	*DREB2D*	Dehydration-responsive element-binding protein	GAACCAAATCGTGGTGCTCG<br>AGCAACATCTTCCACCGTAGG	258
CsaV3_5G028690	*FBA*	Fructose-bisphosphate aldolase	TTGTCCAGACCGCGAAATCA<br>CAGGTGTGGTCAGCAAAAGC	156
CsaV3_5G008880	*FRB*	Fructose-1,6-bisphosphatase	TCCTTCAAAGTCAGGACGGC<br>TATCACCTGGCCAGACTCCA	283
CsaV3_1G039270	*FSD*	Fe superoxide dismutase	TCCACTTTCCTGCTGGCTG<br>GTAAGGATGCCTGGTGCTCT	149
CsaV3_4G023250	*GID1*	Gibberellin-insensitive dwarf	TGGTGTTCTCCGCGTTTACA<br>CAGGTTGTGGATGGGGTCAA	127
CsaV3_7G033380	*GLYR1*	Glyoxylate reductase	TGACACCGCCAAATCCATCA<br>TCACGACTCAGCCAAGAACC	285
CsaV3_3G009450	*GLYR2*	Glyoxylate reductase	ATCCGTCCGAGCTTGTCTTG<br>GAACCGATCTCCCGCTGAAA	250
CsaV3_4G029990	*G6PD*	Glucose-6-phosphate 1-dehydrogenase	TCCCTGGTCTAGGAATGCGA<br>AGCTAAGTAGTGAGCCCCGA	263
CsaV3_7G007260	*GSA*	Glutamate-1-semialdehyde 2,1-aminomutase	GGAATGGCTGGCTCTTTTGC<br>GGGGAATTCACTCCACCAGG	206
CsaV3_2G030530	*HEMA*	Glutamyl-tRNA reductase	CACGGTGGGTAAACGGGTAA<br>ATTCTGGCAGTGGCATGTGA	123
CsaV3_4G008050	*HEME*	Uroporphyrinogen decarboxylase	ACTATTGGTGAAGGCGGCAA<br>GAAAGGAACGCCAAGTGCAG	247
CsaV3_1G032180	*HKT1*	High affinity K+ transporter	GTCGTCATCCACGATGGTCA<br>ACCAAGAACATTCGTCGCCA	109
CsaV3_1G030930	*LAX*	Auxin resistant 1 (AUX1)/Like AUX1 (AUX1/LAX) influx carrier	TCTTGATGATGAGCCACCCC<br>ATCTGAAGGTCAAGCTTACGCA	180
CsaV3_1G004740	*MSD*	Mn superoxide dismutase	AGAAGCTCCCCTGGTTGAGA<br>CTCTCGTGGTCTCACGCATT	200
CsaV3_1G033530	*MPK3*	Mitogen-activated protein kinase	GTCCTCCGATTATGCCTATTGG<br>TCTTAACCGCAACCATTTCGT	90
CsaV3_6G032360	*MPK6*	Mitogen-activated protein kinase	CCGTGCACCAGAGCTCTTACT<br>CAAGGGCTTCCGATCCATTA	103
CsaV3_1G004260	*MPK9*	Mitogen-activated protein kinase	CGTGCTCCCGAACTTTGTG<br>TTCCAGTAAGCATTTCCGCA	97
CsaV3_3G044540	*NAC35*	NAC domain-containing transcription factor	GGTCATCGTCCACGTGTTCT<br>GCCTGAGACTGAGCAAGAGG	250
CsaV3_4G028100	*NAC41*	NAC domain-containing transcription factor	AGGGGGCAATCGAGAAACAG<br>TGAACTCCGATGACACCACG	252
CsaV3_6G034250	*NAC66*	NAC domain-containing transcription factor	GGCGATGTGTTAATGCCGTC<br>TCCTTCCATTTTCGCTCGCT	164
CsaV3_4G007760	*NCED2*	9-cis-epoxycarotenoid dioxygenase	GGCGATGGAATGGTTCATGC<br>ACCGAGCTATCCCAGAGTGT	163
CsaV3_7G018780	*NHX4*	Sodium/hydrogen exchanger	AAAAACATCGGCAGCGATCA<br>TTTTCCCCTTGCTGCAGACC	181

CsaV3_4G027860	*P5CR*	Delta-1-pyrroline-5-carboxylate reductase	GGTTGAGCCGTTACTGTGGA<br>TCCAGCTCCGATGAACCCTA	126
CsaV3_3G034580	*P5CS*	Delta-1-pyrroline-5-carboxylate synthase	CCAAGAATGCAAGGCGTATCG<br>CAACAGCTGCACATGCCTTT	264
CsaV3_7G004050	*PETC*	Cytochrome b6-f complex iron-sulfur subunit	CCATTGGACTTCCCACTGCT<br>TCCATGTGCCTTCAGCCATT	146
CsaV3_3G046150	*PETE*	Plastocyanin	TTCTTACAACCATGGCCGCT<br>ATAGCTTTGGGGATGCAGGG	129
CsaV3_5G038830	*PGD*	6-Phosphogluconate dehydrogenase	GCTGACCTCTCAGTTGCTGT<br>TCCAAATCCGGGCAAGTTCA	283
CsaV3_3G000190	*PIN*	Auxin efflux carrier family protein	CATTTTTGGAGCGGTGGCAT<br>GCTTTCACCTTTGACGACCG	168
CsaV3_7G003750	*PRX27*	Peroxidase	ACAATTTCACCGGCAAAGGC<br>CGTCGAGAAGAGCGGAATCA	201
CsaV3_4G029960	*PRX47*	Peroxidase	GCAGGGGGTCCATTTTACGA<br>TCGTAAAATGGACCCCCTGC	221
CsaV3_6G043930	*PRX73*	Peroxidase	CCTGTGCCGATATTCTCGCT<br>GCCACAGTGTGAGAATCCGA	245
CsaV3_3G013420	*PsaD*	PSI reaction center subunit D	CTCCTTCCTCACCTCCAAGC<br>TTTCAGCAGATTCGGCCCTT	286
CsaV3_2G010090	*PsaE*	PSI reaction center subunit E	ATGGCAATGGCTGCCTCTAA<br>CGGAGGCACGGATAACCAAT	163
CsaV3_1G046680	*PsaF*	PSI reaction center subunit F	AAGCAATTCGCCAAGAGGGA<br>GCACCACAAAGAAGCCCTTG	161
CsaV3_3G025000	*PsaH*	PSI reaction center subunit H	ATTTGGGCAACACCACTGGA<br>CTGGGGCAGTAGCACTGAAA	183
CsaV3_6G052210	*PsaK*	PSI reaction center subunit K	CTCTCAGTGCTCGTTGTGGT<br>TCTGTAGCCCAGAGTCCCTC	165
CsaV3_2G009680	*PsaL*	PSI reaction center subunit L	GAAGGAGAGCCATCTACCGC<br>ACCGGAGATACCACCGAAGA	126
CsaV3_6G041600	*PsaN*	PSI reaction center subunit N	TCTCCTGGGGTTGTGGGTTA<br>CCTTGCGAAGTTTGCTCCAC	249
CsaV3_6G042680	*PsbO*	PSII oxygen-evolving enhancer protein	ACCCAAAGGGAAGAGGAGGT<br>GTCTCAGGCTTGCTCTTGGT	160
CsaV3_5G026270	*PsbP*	PSII reaction center subunit P	CCGCTTTTGTTGGGAGTTGG<br>TGAATGCCGAATCATGGGCT	175
CsaV3_1G011330	*PsbQ*	PSII oxygen-evolving enhancer protein	GGGCTGGTAGTGGAAGCAAT<br>TGGTGCTAGCCACACTCAAG	127
CsaV3_4G007600	*PsbR*	PSII reaction center subunit R	GCCACGTGTCTTCCTACCAA<br>CTCGCCAGAGATGGAAGACC	218
CsaV3_2G004030	*PsbW*	PSII reaction center subunit W	TGCCCACGTTGCAAAAGATG<br>CTCAAACCAAAAGGCAGCCC	205
CsaV3_2G001270	*PsbX*	PSII reaction center subunit X	CGGTGGAGTAGTCGTCACTG<br>ATTTACAGCAGGAGCCGCAT	197

CsaV3_5G030690	*PsbY*	PSII core complex protein Y	CTCTTTTGCTGCCGCTCATC<br>ACAAAACCCAAGCAATCGCC	240
CsaV3_4G026530	*PYL8*	ABA receptor PYR/PYL family	GGGAGGAGACAGCATATCTAACC<br>GGAGTGGGTTATGCGATGGG	139
CsaV3_5G034360	*RBCS*	Ribulose bisphosphate carboxylase small chain	CGTGTACCGTGAGAACCACA<br>AATCTTGGGGGCTTGTAGGC	219
CsaV3_5G008270	*RCA*	Ribulose bisphosphate carboxylase/oxygenase activase	ACCCCACCAATGTCCAACTC<br>GACACCGATACGGTCTTCCC	167
CsaV3_7G029990	*RGL2*	DELLA protein	ACACCATTTGATTTCAGTGGCTC<br>TCTTTCCCTCGTTGCTCTGTC	95
CsaV3_6G046510	*SAMT*	salicylic acid carboxyl methyltransferase	CGTGGTGGACGAATGGTTGT<br>TTCCACAATCCCCTCAGCAA	129
CsaV3_6G039090	*SGAT*	Serine-glyoxylate aminotransferase	TTCCCTTCCCACCGGAATTG<br>TCCAAAGCTGCTCTAAGCCC	183
CsaV3_5G017250	*SOS1*	Na^+/H^+ antiporter	CGGTAGCATGGTTGATTTTCG<br>GATTCGACCGGCTATGAGATG	80
CsaV3_4G027060	*SOS2*	CBL-interacting protein kinase	TGTGGAACCCCTGCTTATGTC<br>CGCACGACCAAATATCAGCTT	82
CsaV3_6G048120	*SOS3*	Calcineurin B-like (CBL) protein	CAAGGAAGAGTGGCGAAACC<br>TGGGAACGTGGTCGTGATATC	94
CsaV3_6G006270	*TKL*	Transketolase	GCTGAGAAGCATTTAGCCGC<br>ACATGCCACCCTAAAGCCTC	251
CsaV3_1G001640	*TPS*	Trehalose-6-phosphate synthase	GCACCTGACTTTGTGCTGTG<br>AGCAGGGTCAAAACATCGGT	185
CsaV3_3G007160	*WRKY18*	WRKY family transcription factor	AGCGATGTTGATGTGCTGGA<br>GTAAAGGGTTCGTGCTCCGA	233
CsaV3_3G008610	*WRKY21*	WRKY family transcription factor	TCCAAACGCCGGAAGAATCA<br>CTTGTTTTCGGGCCATGCAA	172
CsaV3_6G028510	*WRKY46*	WRKY family transcription factor	GCAAGCCAAAACCAACGAGT<br>GGACAACGAAAACGTGGTGG	236

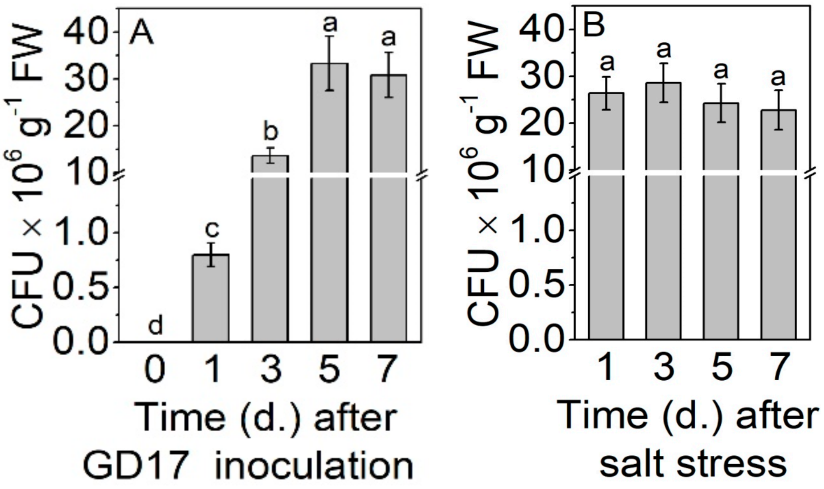

Figure S1. Colonization efficiency of GD17 inside roots as indicated by colony-forming units (CFU). GD17 inoculation was performed at 7 days after seed germination (A) and salt stress was carried out 7 days after inoculation (B). The data were collected from three replicated experiments (n = 3) with 10 plants used in each batch of experiment, and results are presented as means ± SD. Bars labeled with different lowercase letters indicate significant differences at $P < 0.05$.

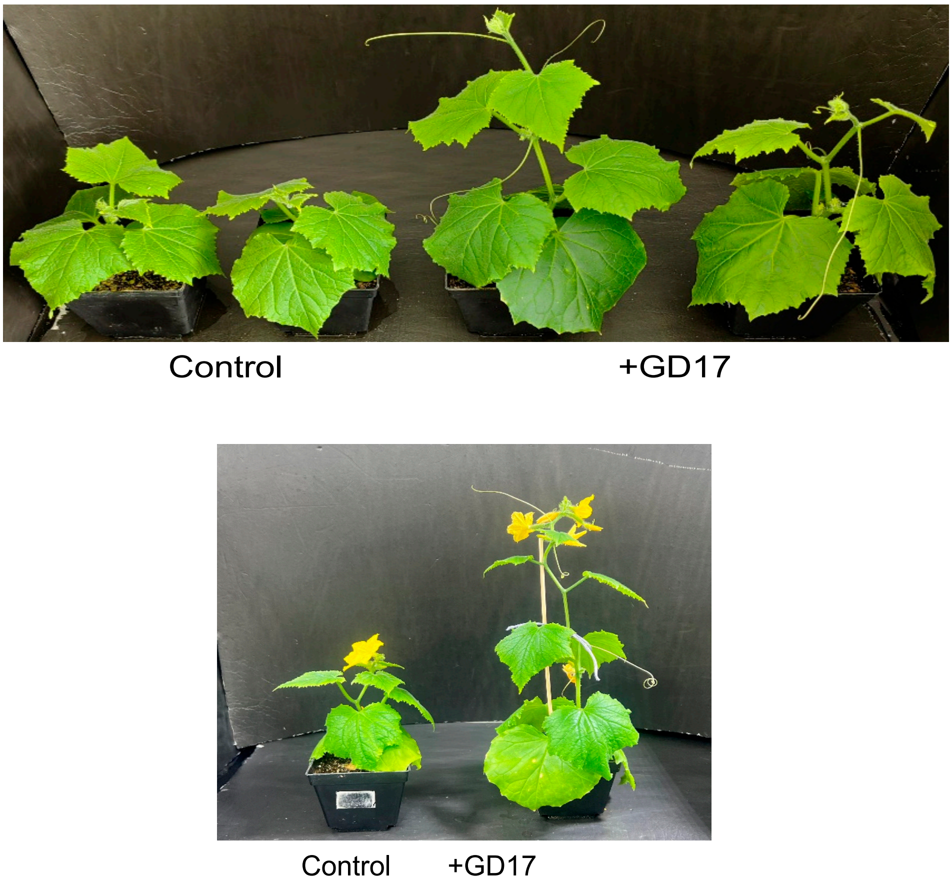

Figure S2. Representative photographs illustrating the effect of GD17 inoculation on plant development. Seven-day-old seedlings were inoculated with GD17. After 28 days of inoculation (upper panel) and 48 days (lower panel), the developmental status was compared between inoculated plants and non-inoculated controls.

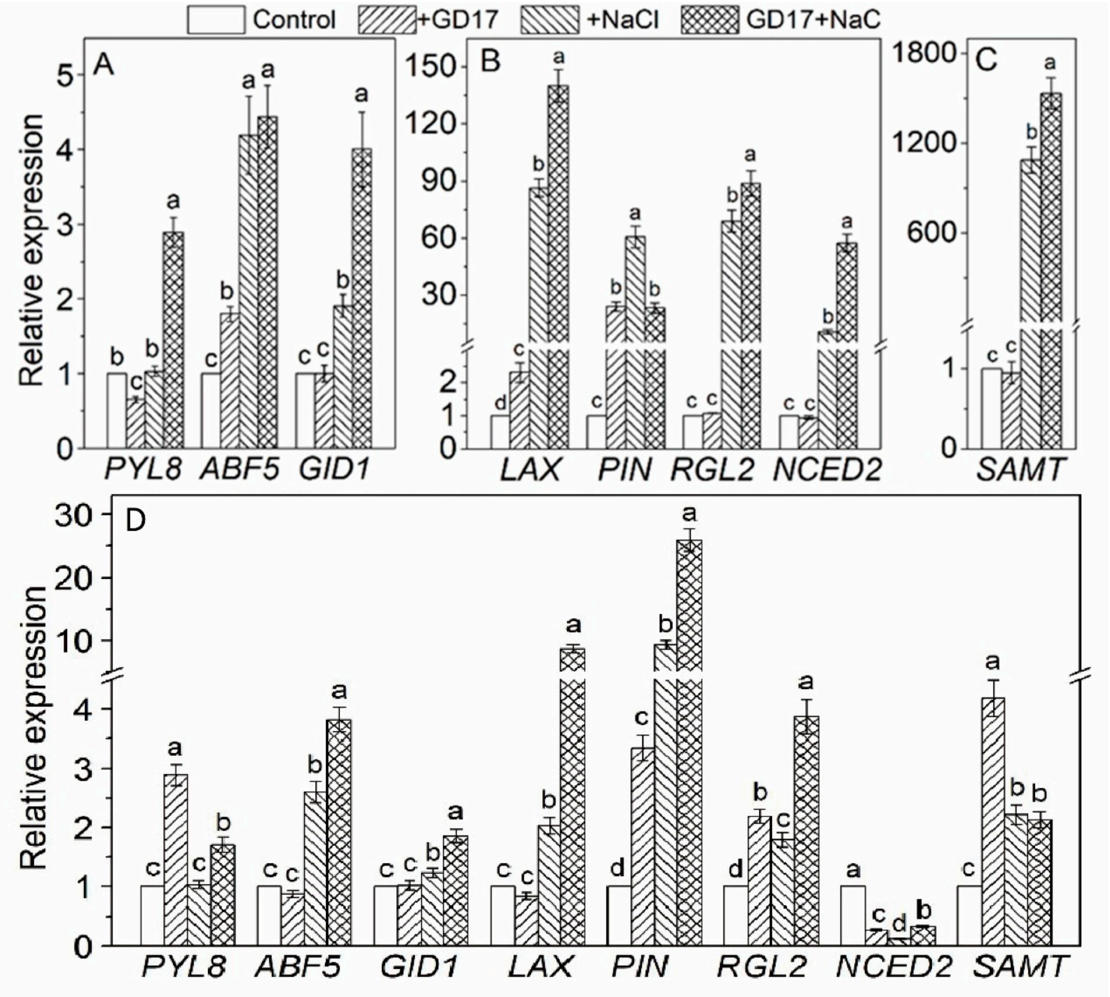

Figure S3. Effect of GD17 inoculation and/or salt stress on the expression of phytohormone synthesis and signaling-related genes in plant leaves (A-C) and roots(D). Data are presented as means ± SD from three independent experiments (n=3), with 20 seedlings per experiment. Different lowercase letters indicate significant differences at $P < 0.05$.

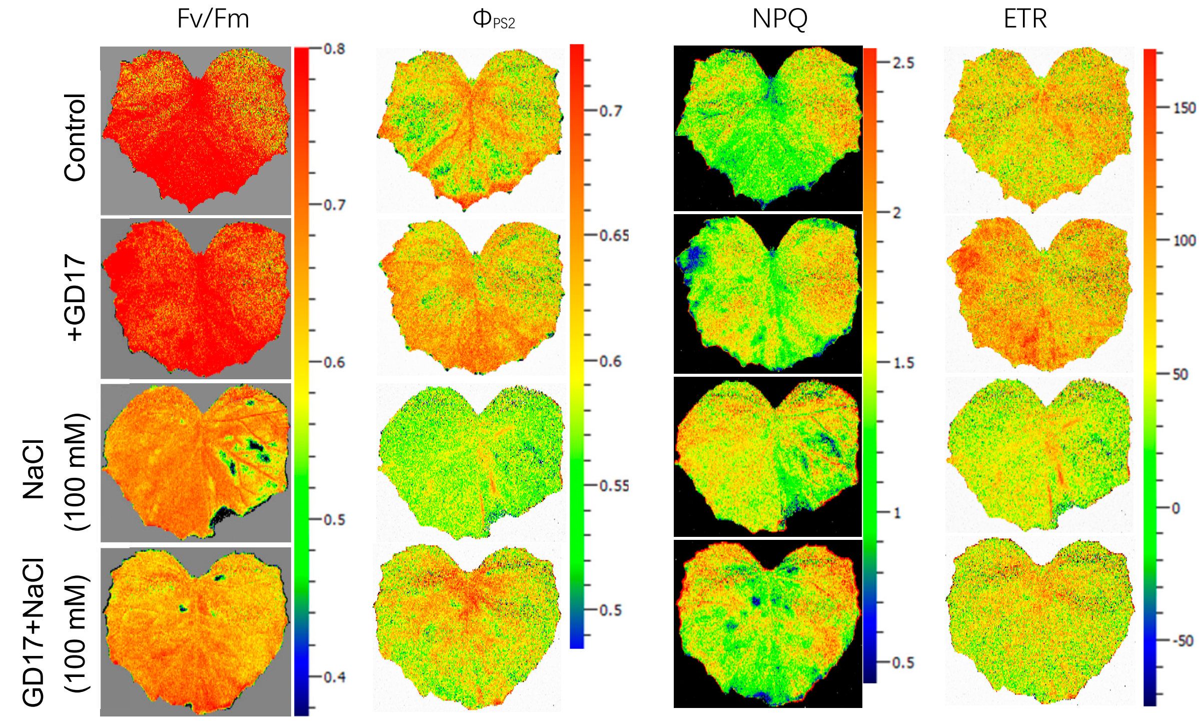

Figure S4. Representative pictures of chlorophyll a fluorescence imaging. Fv/Fm, maximum quantum efficiency of PSII; ΦPSII, actual quantum efficiency of PSII; NPQ, non-photochemical fluorescence quenching; and ETR, electron transport rate.

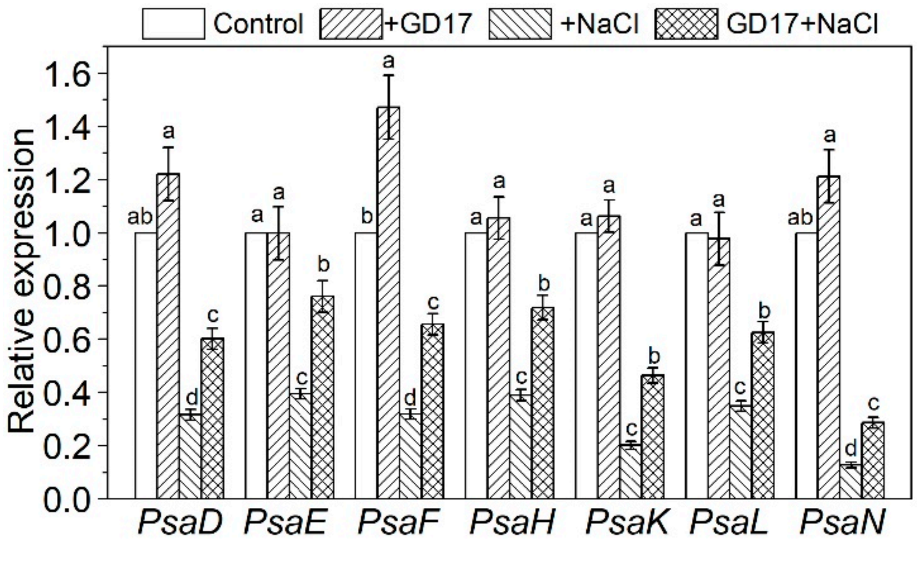

Figure S5. Effect of GD17 inoculation and/or salt stress on the expression of PSI-related genes. Data are presented as means ± SD from three independent experiments (n=3), with 20 seedlings per experiment. Different lowercase letters indicate significant differences at $P < 0.05$.

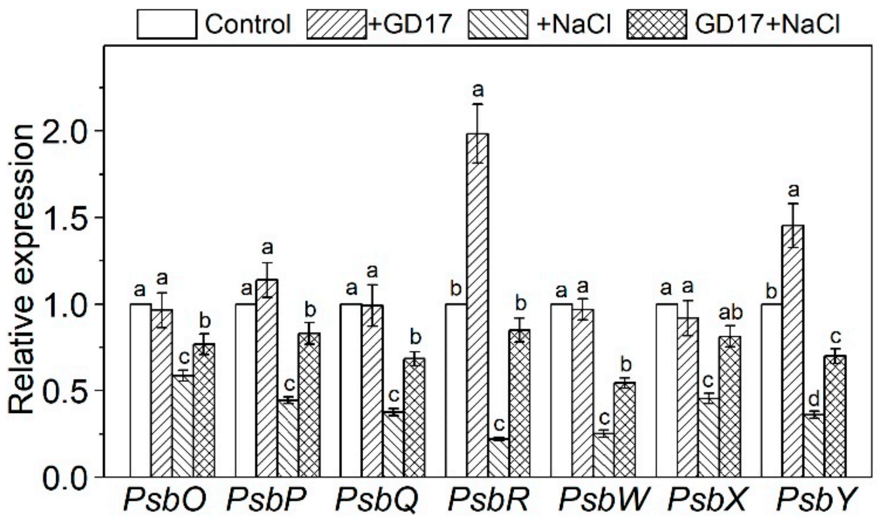

Figure S6. Effect of GD17 inoculation and/or salt stress on the expression of PSII-related genes. Data are presented as means ± SD from three independent experiments (n=3), with 20 seedlings per experiment. Different lowercase letters indicate significant differences at $P < 0.05$.

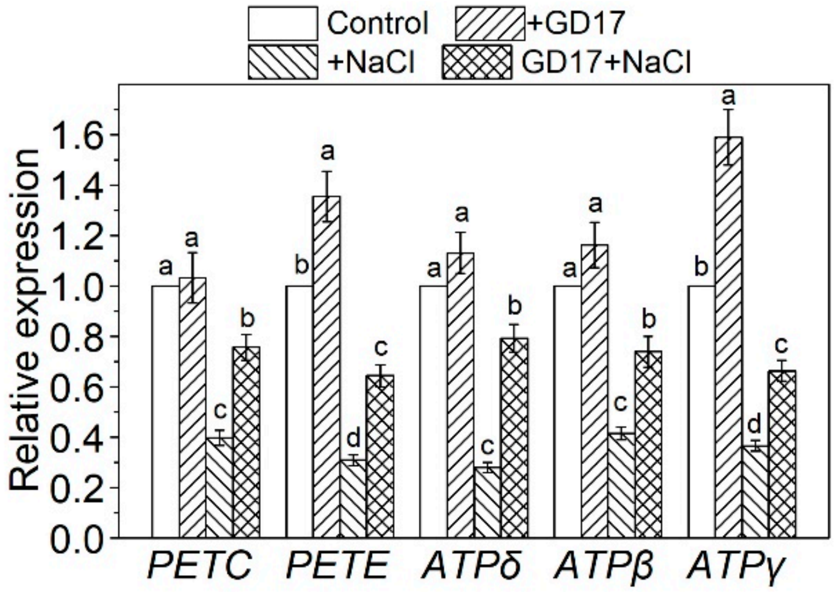

Figure S7. Effect of GD17 inoculation and/or salt stress on the expression of genes related to the electron transport and photophosphorylation. Data are presented as means ± SD from three independent experiments (n=3), with 20 seedlings per experiment. Different lowercase letters indicate significant differences at $P < 0.05$.

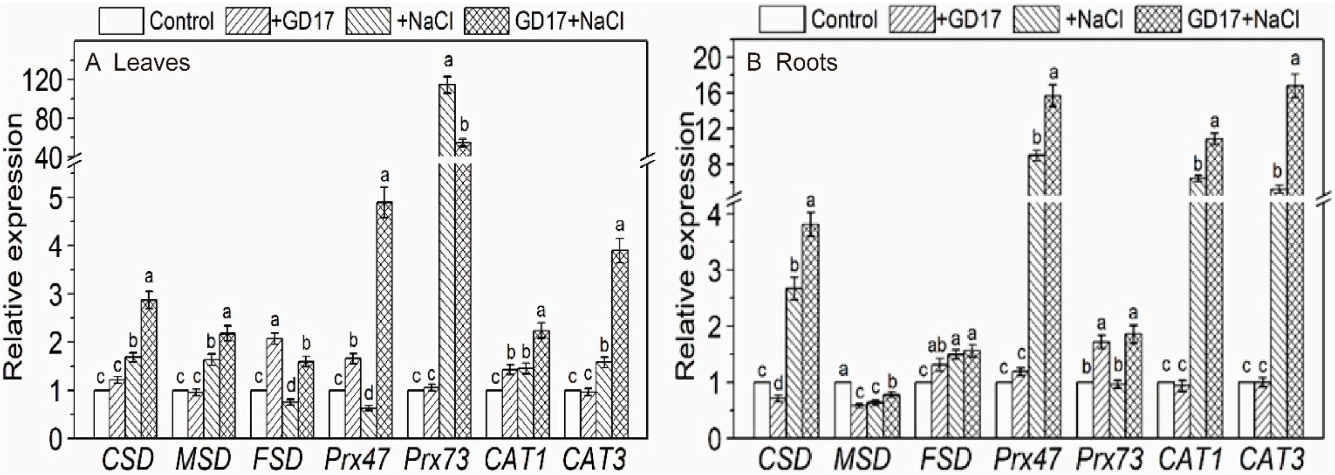

Figure S8. Effect of GD17 inoculation and/or salt stress on the expression of antioxidase-related genes in plant leaves (A) and roots (B). Data are presented as means ± SD from three independent experiments (n=3), with 20 seedlings per experiment. Different lowercase letters indicate significant differences at $P < 0.05$.
